# The "Pall Mall Gazette" and the London Hospital

**Published:** 1893-07-29

**Authors:** 


### The Hospital

AN INSTITUTIONAL JOURNAL OF

Science, Medicine, Nursing, and Philanthropy.

*For Hospitals, Asylums, Infirmaries, Dispensaries, Medical Practitioners, Students, Nurses, and the Charitable Public.*

VOL. XIV.—No. 357.] JULY 29, 1893.

### THE "PALL MALL GAZETTE" AND THE LONDON HOSPITAL.

THE new editor of the *Pall Mall Gazette* is a cultured gentleman and a Member of Parliament. Does he find his parliamentary duties so onerous as to compel him to leave most of his editorial duties to others? This would seem to be the case, for we cannot account for his treatment of the London Hospital in any other way. He has attacked the London Hospital in three articles, which he says were written in response to a challenge in THE HOSPITAL. Now this is a statement of fact, and it is either true or not true. We stated last week in THE HOSPITAL that it was not true. We repeat our statement to-day, and we challenge the editor of the *Pall Mall Gazette* to refute it. The words we used last week were these: "Can our contemporary the *Pall Mall Gazette* have been hoaxed? . . . It certainly looks like it, for the article"—that is the first of the three articles in the *Pall Mall Gazette*—"states 'that having read the appeal' which appeared in THE HOSPITAL of June 3rd last, the writer 'resolved to enter the London Hospital as a nurse.' That is she resolved to enter, did so enter, and has written her article as the result of the information so obtained. Now this article purports to be a description of many parts of the hospital and its management, and makes statements which could not possibly be learned by a paying probationer of a few days' residence. But if it be true that our appeal induced the writer to go as a paying probationer to the London Hospital, she could not have entered it earlier than July 3rd, for it appears from inquiries we have made that no paying probationer has been admitted to the hospital between the date when our first article appeared (June 3rd), and the present date (July 19th), with one exception. The paying probationer who constitutes the exception entered the hospital on July 3rd last. It is therefore important to ask the editor of the *Pall Mall Gazette* to promptly publish the date when his special commissioner became a paying probationer at the London Hospital, and the date when she left it." To be quite plain we repeat our denial of the truth of the *Pall Mall Gazette's* statements of fact on documentary evidence in our possession. We push it home to the editor that there has been some un-English quibbling at work. In accepting the articles of what he calls his "special commissioner" he has been disloyal to the ordinary traditions of editorship. Either he does not understand those traditions, or he has chosen to wilfully disregard them. Either he has not taken the trouble to find out whether his "special commissioner's" articles were *bonâ fide*, that is real inquiries instituted and carried out in response to and after our challenge, or he has deliberately connived at a misstatement of a plain matter of fact. In the *Pall Mall Gazette* of the 26th inst. we have further evidence of the un-English character of this attack on the London Hospital, as a number of letters are published, all from anonymous correspondents, all hostile to the hospital, and several of which were published long ago in other journals.

We purposely avoid beating about the bush. It is our object to compel the editor of the *Pall Mall Gazette* either to frankly clear up the points we have raised or to accept the odium of being an enemy of our greatest London medical charity, who has permitted his editorial position to be used to gratify the spleen of other persons whom he particularly wishes to oblige. These things are said deliberately. The editor of the *Pall Mall Gazette* has permitted a false statement to appear in the paper of which he is the responsible conductor, and when the falseness of the statement has been pointed out to him he has taken no step to correct the statement or to undo the mischief it may have caused. In our judgment, speaking as we do, after a most careful testing of every statement they contain, the series of articles which have appeared in the *Pall Mall Gazette* upon the London Hospital are among the most disgraceful, wanton, and aggravated libels which have ever appeared in an English newspaper.

The London Hospital has had put upon it the task of convincing the editors of newspapers, and through them the public, that the seventy or eighty thousand a year, which the public commits to its care, is spent honestly, economically, and in the best interests of patients, nurses, and the whole profession and practice of the healing art. There can be no difficulty in this. There are hosts of witnesses who know all about the hospital. Its physicians and surgeons are numbered by the dozen; its nurses and its students by the hundred; its patients by the thousand. Moreover, the hospital possesses documentary evidence which proves beyond doubt that it is an admirably managed institution, and that every charge of any importance made by the "special commissioner" of the *Pall Mall Gazette* is absolutely and diametrically the opposite of truth. The work of the hospital is not done down in a well. It is done in the centre and heart of London; and the doors of the hospital stand open night and day for any casual passer-by to enter and investigate for himself. Under these circumstances, there cannot be the slightest difficulty in placing such an array of facts before the editors and the public of the metropolis as will set the question of the hospital's merits at once and permanently at rest.

The other voluntary hospitals of London and the country must not think they can hold themselves aloof from the fight. It is, we believe, by design and not by accident that the London happens to be the particular hospital to be assailed. But the enemies of hospitals have tasted blood; and apparently the taste is to their liking. Moreover, they appear to have gained the ears of some newspaper editors. All this means mischief. There is no reason to suppose that these firebrands have any definite plan of campaign, or that they have any present evil designs against hospitals in general. But there is abundant evidence to show that they have found out how to make use of hospitals as levers for the accomplishment of their own private ends. There is evidence, also, to show that they are not at all squeamish. They are something like Cleopatra, quite willing to wreck the whole hospital kingdom so long as their personal wishes are abundantly gratified. There are plenty of modern Neroes, both among men and women, who could fiddle quite gleefully if they saw Rome burning. All the hospitals must take these facts to heart. The renewed attack upon the London Hospital is a challenge to them all to set their houses in order. Perhaps at this moment there may be a dozen innocent-looking paying probationers preparing squibs and crackers for their various evening newspapers on the lines of the *Pall Mall's* special commissioner.

The demand of the moment is action—intelligent, determined action. The committee of the London Hospital, either with or without the sanction of a special meeting of governors, must take proceedings at once. The *Pall Mall Gazette* must be compelled either to do public justice to the institution it has so maliciously traduced, or to pay in solid thousands for the unjustifiable and unparalleled injury it has wrought. The London Hospital is not a shuttlecock to be tossed about between contending parties. It is an army corps maintained for the carrying on of continuous warfare against the sickness and disease of one of the most populous parts of Great Britain. Its serious paralysis would be an imminent danger to the metropolis and the country; its partial ruin cannot be contemplated without dismay. Its defenders must be up and doing without the delay of a moment. Besides, there are the enemies to punish as well as to conquer. We do not believe in vindictiveness; but we do believe in the unfailing punishment of wilfully injurious persons. If public institutions have at present no means of punishing their defamers, new means must be devised. A progressive civilisation is equal to the production of new resources of punishment. If it is not, it will not long be a progressive civilisation. In the spirit of the warlike Hebrews who carried victory to annihilation, the London Hospital must leap to arms, and never call halt until it has "set its foot upon the necks of its enemies."

